# Energy loss associated with *in-vitro* modeling of mitral annular calcification

**DOI:** 10.1371/journal.pone.0246701

**Published:** 2021-02-16

**Authors:** Philip C. Wiener, Ahmed Darwish, Evan Friend, Lyes Kadem, Gregg S. Pressman

**Affiliations:** 1 Division of Cardiology, Heart and Vascular Institute, Einstein Medical Center, Philadelphia, PA, United States of America; 2 Department of Mechanical, Industrial and Aerospace Engineering, Concordia University, Montreal, Canada; Cincinnati Children’s Hospital Medical Center, UNITED STATES

## Abstract

**Introduction:**

Study aims were to compare hemodynamics and viscous energy dissipation (VED) in 3D printed mitral valves–one replicating a normal valve and the other a valve with severe mitral annular calcification (MAC). Patients with severe MAC develop transmitral gradients, without the commissural fusion typifying rheumatic mitral stenosis (MS), and may have symptoms similar to classical MS. A proposed mechanism relates to VED due to disturbed blood flow through the diseased valve into the ventricle.

**Methods:**

A silicone model of a normal mitral valve (MV) was created using a transesophageal echocardiography dataset. 3D printed calcium phantoms were incorporated into a second valve model to replicate severe MAC. The synthetic MVs were tested in a left heart duplicator under rest and exercise conditions. Fine particles were suspended in a water/glycerol blood analogue for particle image velocimetry calculation of VED.

**Results:**

Catheter mean transmitral gradients were slightly higher in the MAC valve compared to the normal MV, both at rest (3.2 vs. 1.3 mm Hg) and with exercise (5.9 vs. 5.0 mm Hg); Doppler gradients were 2.7 vs. 2.1 mm Hg at rest and 9.9 vs 8.2 mm Hg with exercise. VED was similar between the two valves at rest. During exercise, VED increased to a greater extent for the MAC valve (240%) versus the normal valve (127%).

**Conclusion:**

MAC MS is associated with slightly increased transmitral gradients but markedly increased VED during exercise. These energy losses may contribute to the exercise intolerance and exertional dyspnea present in MAC patients.

## Introduction

Mitral annular calcification (MAC) is commonly encountered in clinical practice. It is associated with calcific aortic valve disease, older age, and renal insufficiency [1–5]. On occasion, it causes severe mitral stenosis; more commonly it results in a small resting gradient across the mitral valve [6–9]. While we know of its association with cardiovascular events little is known about its effects on transport of blood through the heart and how these effects are expressed clinically [10–12]. Studying MAC physiology *in vivo* is difficult as affected patients frequently have multiple comorbidities, which act as confounders, and are often unable to exercise. Therefore, to isolate MAC as a variable, we created a three-dimensional (3D) MAC model for *in vitro* testing. MAC phantoms were incorporated into a 3D-printed normal mitral valve derived from transesophageal echocardiographic (TEE) data that was then tested in a cardiac duplicator equipped to measure transvalvular gradients. Particle imaging velocimetry was used to measure viscous energy dissipation (VED) in the left ventricle and comparisons were made with a 3D-printed normal valve (control) which had no MAC. In this way, we were able to isolate effects of MAC on hemodynamics and energy loss as a blood analogue traversed the mitral valve into the left ventricle and was pumped out through the aortic valve. Our hypothesis was that VED may detect abnormalities in the efficiency of blood transport through the heart that are not reflected in standard transvalvular gradient measurements.

## Methods

### 3D patient-specific MAC model

**Fig 1:**
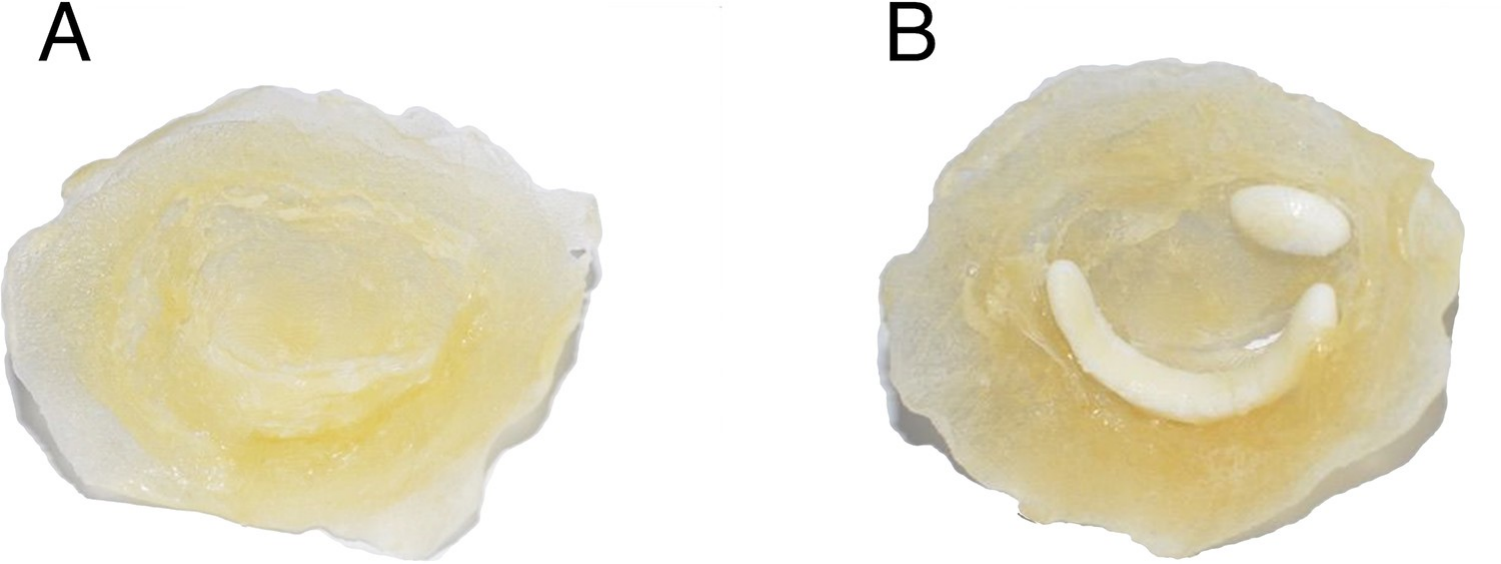
Mitral valve in surgeon’s view as recreated from transesophageal echocardiography and 3D-printing. (A) Normal mitral valve without mitral annular calcification and (B) with calcium phantoms encompassing the posterior annulus and at the base of A3.

**Fig 2:**
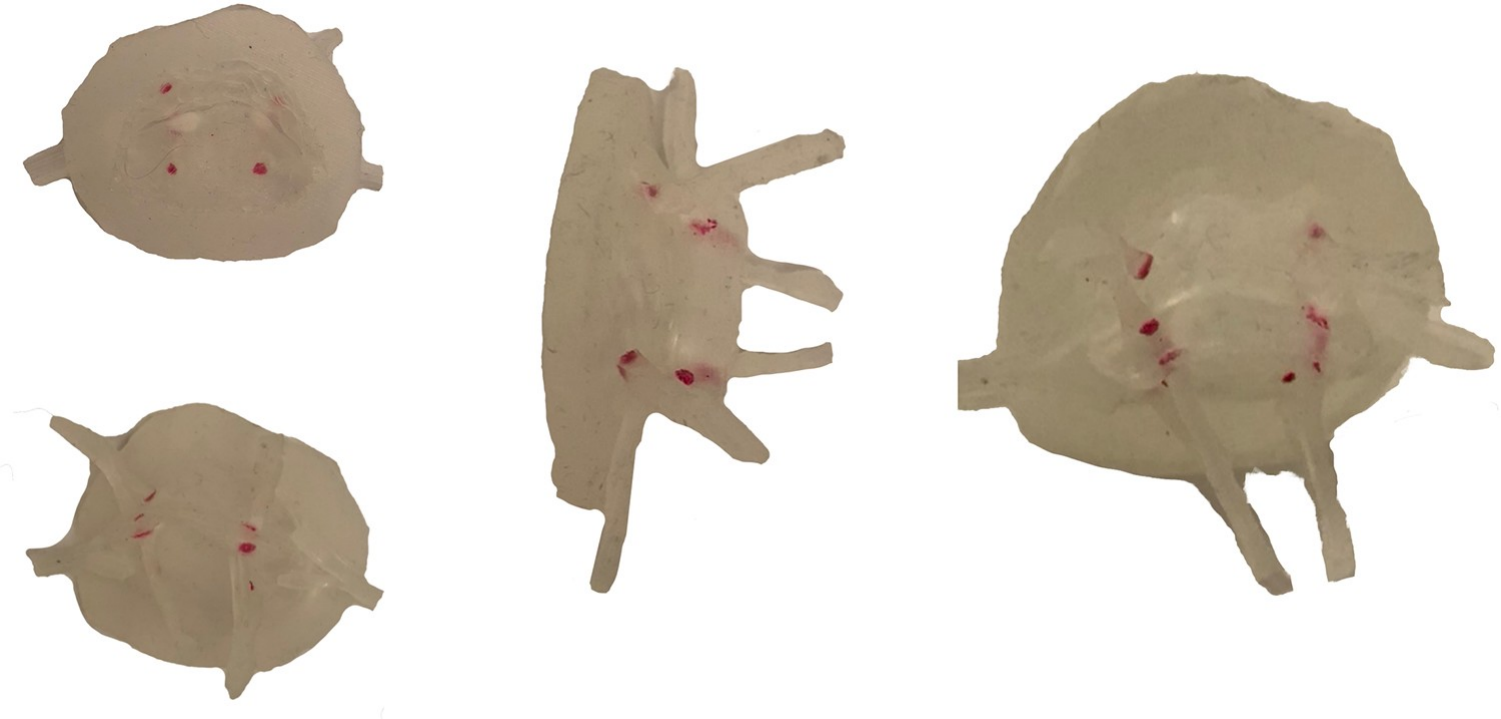
3D printed mitral valve model. Multiple views of the mitral valve model with chordae, prior to being sewn into the left ventricle.

A 3D-dataset from transesophageal imaging of a normal mitral valve was used to create a silicone model for in vitro testing following ethical approval by the Einstein Healthcare Network IRB. IRB granted a waiver of consent as the dataset was previously de-identified. The dataset was acquired with a Philips iE33 echocardiograph and X7-2T transesophageal probe. Using Philips QLAB (Bothell, WA, USA) we manually determined the frame where the mitral leaflets began coaptation. This allowed for optimal visualization of the entirety of both leaflets with minimal dropout due to MAC. We believed this leaflet position would also allow the valve to seal in systole without adding significant resistance to flow in diastole. Data were then converted from standard 3D-DICOM to Cartesian format once more using QLAB. Next, the Cartesian data was converted to a specific format for 3D-segmentation in ITK-SNAP version 3.8.0 [13]. The volume enclosed by the mitral valve and annulus was segmented thereby allowing for a “negative” 3D scaffold on which annulus and leaflets could be created from silicone. The scaffold was printed using polylactic acid (PLA) on an Ender 3 printer (Creality, Shenzhen, Guangdong, China). The MV annulus and leaflets were created by painting three even coats of Smooth-on Ecoflex 00–20 (Smooth-On Macugie, PA) on the 3D-printed MV scaffold. Afterward, application molds were placed into a 40°C incubator for 2 hours to dry completely. MAC phantoms were created via 3D-printer, again using PLA, and placed so as to simulate severe MAC–one phantom represented the “bar of calcium” often seen encompassing the posterior annulus and a smaller “nodule of calcium” was located at the base of A3 (Fig 1). These were incorporated into the silicone valve print by gluing them in place on the atrial side of the annulus and then painting one additional silicone layer on top. Chordae were subsequently created from strips of silicone which were set in linear molds using the aforementioned Smooth-on Echoflex 00–20) and trimmed to a length of 22mm. The chordae were then split longitudinally roughly 2/3^rd^ of their length and attached to the mitral valve leaflets using cyanoacrylate (“super glue”). Two chordae were attached to each leaflet with an additional two “commissural” chordae attached to either side (anterior and posterior) of each commissure (Fig 2).

**Fig 3:**
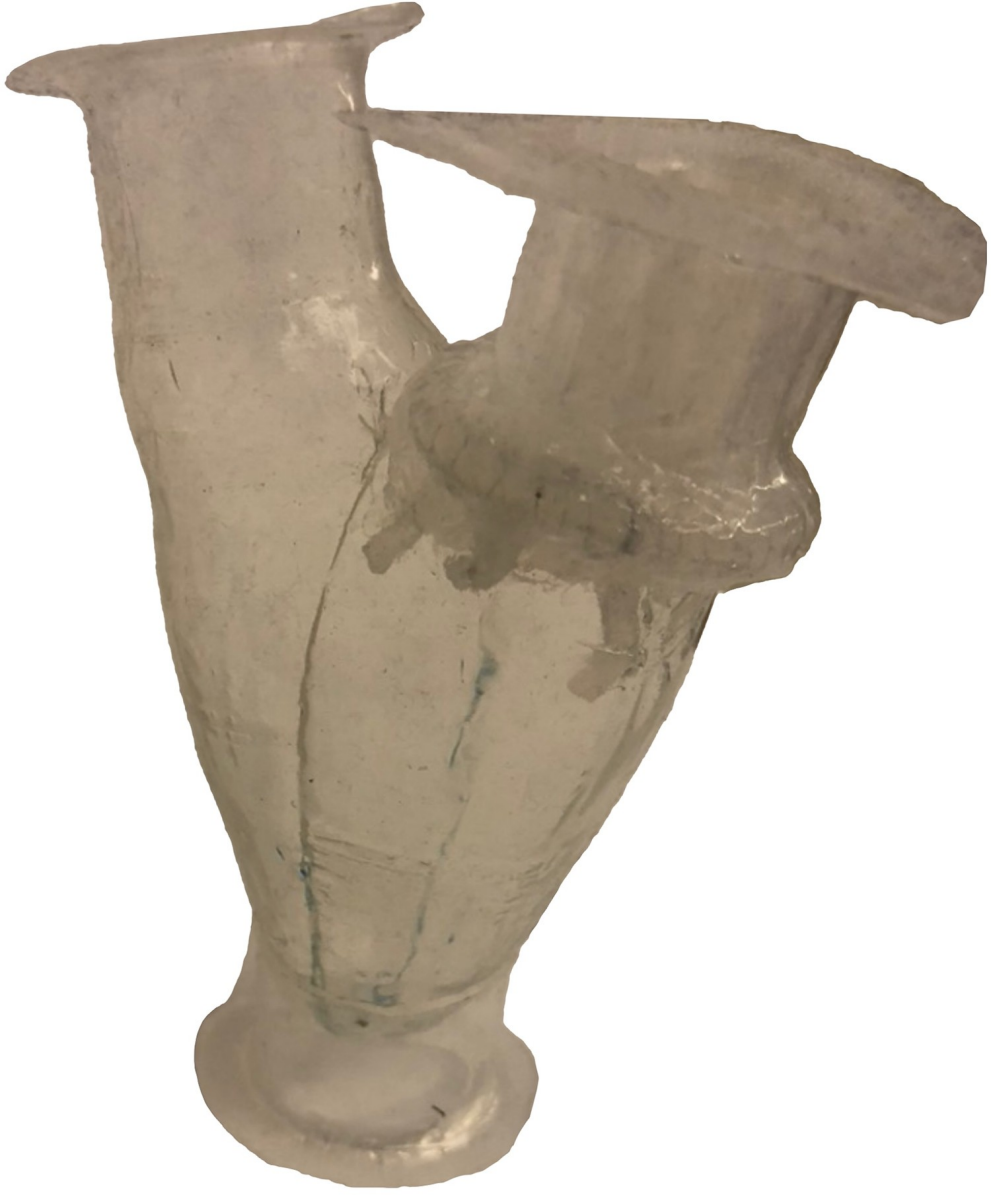
Left ventricular model. Completed left ventricular model with mitral valve sewn into the ventricle inlet, attached chordae, and silicone sealed layer.

An in-house left heart duplicator was used to generate physiological flow conditions inside fabricated left heart chambers (left atrium, left ventricle, and aorta). The left atrial 3D base model was acquired from a 3D construction of a complete adult human heart using CT and MRI scans (Solid Heart Gen 2 Zygote: American Fork, UT, USA). This left atrial base model has accurate orientation of the pulmonary veins along with an appendage to ensure realistic flow conditions. A symmetric left ventricular 3D base model was used (with aorto-mitral angle of 28°) in order to facilitate 2D velocity measurements via particle image velocimetry. The aortic 3D base model has a diameter of 30 mm and includes Sinuses of Valsalva. The three base models (aortic, ventricular, and atrial) were printed in PLA (Polylactic Acid) using a desktop 3D printer (LulzBot TAZ 6, FAME 3D, ND, USA) with print volume dimensions (280 x 280 x 250 mm). Models of the chambers were made by painting transparent silicone (XIAMETER RTV-4234-T4, Dow Corning, MI, USA) over the PLA base models. Each model consumed 5 to 6 layers of material where, after adding each layer, the model was cured while rotating continuously to ensure homogenous silicone distribution. The resulting thickness ranged between 1.5 and 2.5 mm. The left ventricle was created with an inlet and an outlet. The inlet was severed, the mitral valve was sewn onto it by creating a 1–2 mm prolapsed lip (simulating the mitral annulus), and four cardinal sutures were used to maintain position while sewing. Next, the body of the ventricle was similarly attached with four cardinal sutures to the newly fashioned annulus. A running suture was used to adhere the three layers together. The chordae were sewn onto the ventricle with a single loop per chord in near-anatomic positions with minimal tension so as to not tether the valve open. Through developmental testing, the regurgitation was effectively eliminated. Lastly, a thin layer of silicone (Smooth-on Ecoflex 00–20) was painted along the suture line outside of the ventricle and over the sewing puncture points to create a watertight seal (Fig 3).

**Fig 4:**
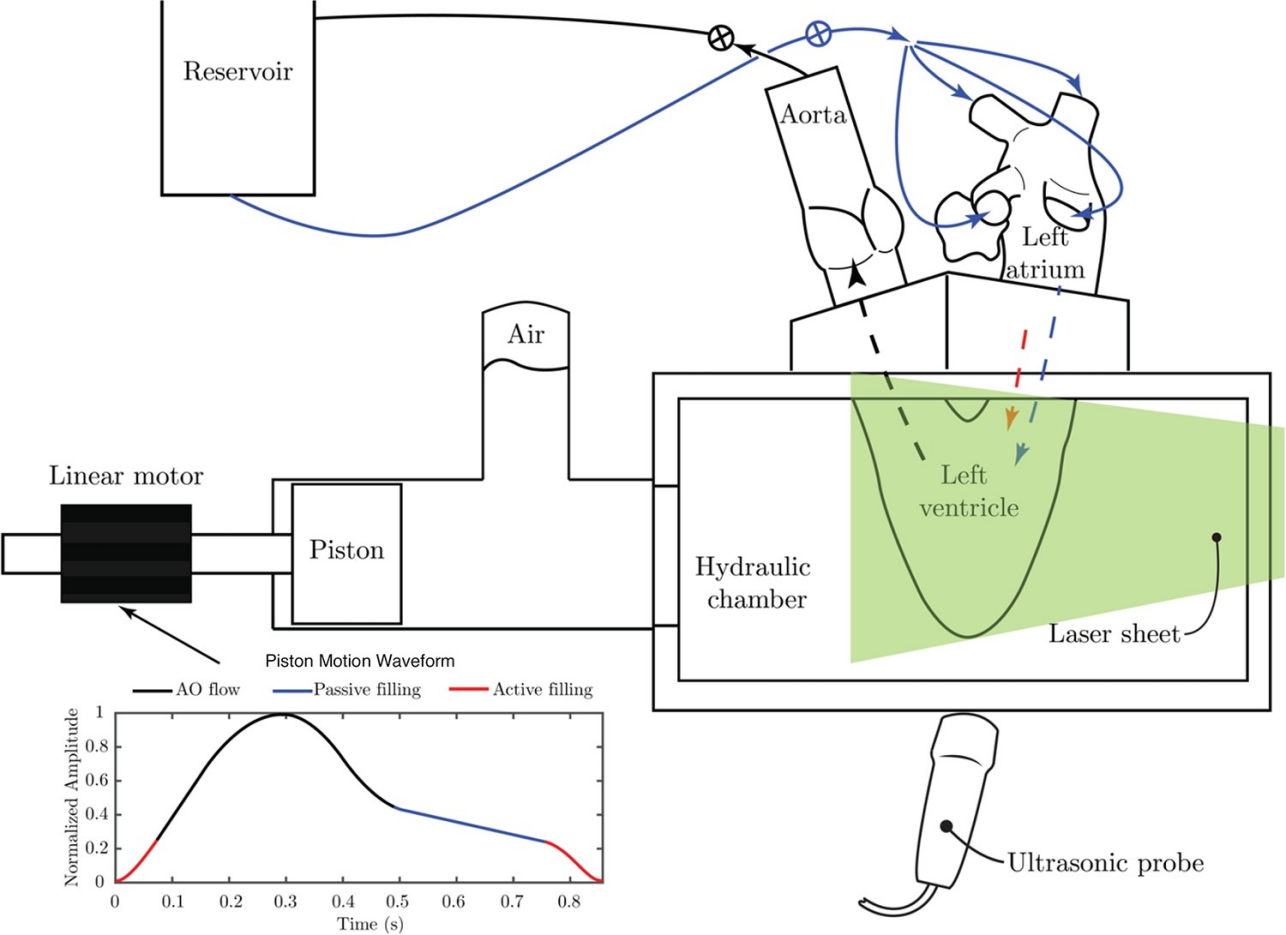
Schematic of the left heart duplicator showing the waveform that governs the forward and backward movements of the piston (by the linear motor). The black portion of the curve represents aortic related flow, while the blue and red portions of the curve represent the passive and active filling of the ventricle, respectively. The arrangement of the cardiac chambers over the duplicator are shown where the left atrium receives flow from a reservoir through four pulmonary veins (solid blue arrows); flow from the atrium into the ventricle occurs in two waves (passive and active) represented by the dashed blue and red lines. The ventricle ejects the flow through the aorta towards the reservoir following the dashed black line. Two resistance valves are attached at the reservoir inlet and outlet to control flow and pressure values.

Cardiac flow was generated by hydraulic activation, wherein a linear motor drives a piston connected to a Lucite hydraulic activation chamber containing the left ventricle. As the motor moves forward, the left ventricle experiences contraction allowing ejection through the aortic valve; reverse movement of the motor induces ventricular expansion causing closure of the aortic valve and filling of the left ventricle. In this experiment, both E and A waves were generated by the expansion of the ventricle similar to what was done by Okafor et al [14]. For more details about the heart duplicator see reference.^15^ A mixture of water and glycerol (60% water by volume; mixture’s dynamic viscosity = 4.2 cP, density = 1100 kg/m^3^) was used as a blood analogue. Left atrial pressure and left ventricular pressure were simultaneously measured using two fiberoptic pressure sensors (FISO FOP-M260, Canada; range -300 to 300 mmHg; accuracy ±1 mm Hg). Doppler gradients were measured using a Logiq E echocardiograph (GE Ultrasound, Milwaukee, WI). The simulator, laser, camera, and echocardiograph were set up as described in Fig 4. Each valve was tested under conditions simulating rest (heart rate 70 beats per minute with stroke volume of 60.08 ml) and exercise (heart rate of 110 beats per minute with stroke volume of 90.13 ml).

**Fig 5:**
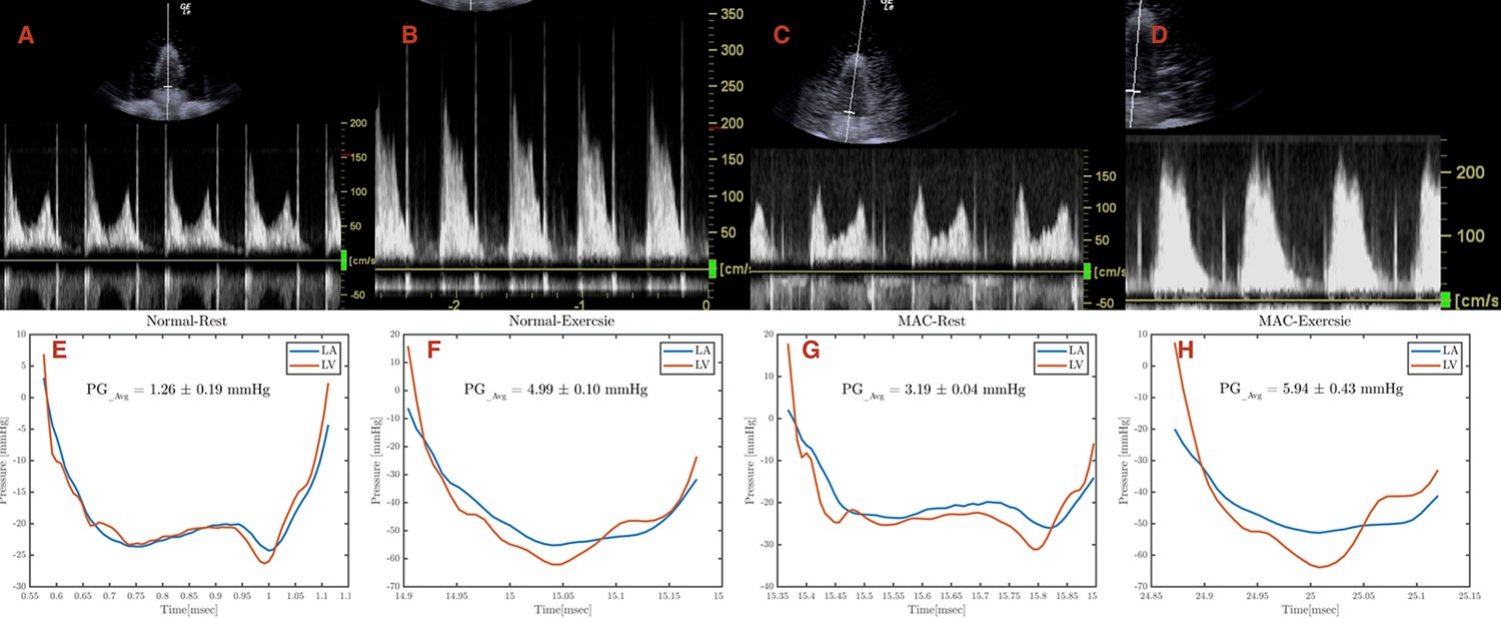
Echocardiographic and catheter assessment of pressure gradients. Continuous wave Doppler measurements of each experimental condition: (A) normal valve at rest, (B) normal valve during exercise, (C) mitral annular calcification (MAC) valve at rest, and (D) MAC valve during exercise. Catheter based pressure measurements of each experimental condition: (E) normal valve at rest, (F) normal valve during exercise, (G) mitral annular calcification (MAC) valve at rest, and (H) MAC valve during exercise.

Doppler gradients were obtained by positioning the imaging transducer under the LV apex and directing the steerable CW Doppler beam through the MV (Fig 5A–5D–top row). The resulting velocity curves were then integrated in standard fashion to obtain the transmitral gradients. Simultaneous catheter-based pressure measurements were made within the atrium and ventricle and recorded every 4 msec. By integrating the difference between the two pressure signals, we were able to compute the average pressure gradient across the valve (Fig 5E–5H–bottom row).

The flow field inside the left ventricle was acquired by time-resolved planar particle image velocimetry (TR-PIV) measurements. To perform this analysis, the water/glycerol mixture was seeded with polyamide particles (mean diameter: 50 μm, density: 1030 kg/m^3^). The particles were illuminated using a double-pulsed Nd YLF laser with a 10 mJ output energy at 1 kHz, 527 nm wavelength, and a repetition rate range between 0.2 and 2000 Hz (Litron Laser, Warwickshire, UK). A plane of laser light was directed through the midportion of the ventricle. Images of the particles were recorded using a Phantom V9.1 high speed camera (1000 fps at a resolution of 1632 × 1200 pixels, Vision Research, Inc., Wayne, NJ, USA). The velocity vectors were computed from the raw images using DaVis 7.2 software (LaVISON GmbH, Göttingen, Germany) and multiple pass fast Fourier transform cross correlation with an initial 64 × 64-pixel interrogation window and a final 16 × 16-pixel interrogation window with a 50% overlap. From the computed velocity fields, VED was calculated as the simulated blood transited across the MV and through the LV.

For this research we used a single model of a normal valve (control), validating its VED by comparison with prior studies using either a bioprosthetic valve [15] or a hydrogel based valve [16], and a single MAC valve. Results were then compared with those of a single MAC valve constructed as described above. Valve areas were calculated using the Gorlin equation: Mitral Valve Area = (0.85) *[Cardiac Output/ (44.3*Heart Rate*Flow Period*√Mean Pressure Gradient)]. Testing was performed 5 times for each valve under resting conditions and 5 times under exercise conditions.

## Results

S1 and S2 Videos show the apparatus in action. Table 1 notes the results of experimental hemodynamic measurements for normal and severe MAC valves at rest and exercise conditions. For the normal valve, the calculated mitral valve area (MVA) was 2.2 cm^2^ (using a diastolic flow period of 0.465 sec). For the severe MAC valve, the MVA was 1.4 cm^2^.

**Table 1:** Experimental parameters for normal valve versus MAC valve.

Table 1: Experimental Parameters for 3D Printed Valves				
	Rest		Exercise	
Parameters	Normal	Severe MAC	Normal	Severe MAC
MVA (calculated, cm^2^)	2.2	1.4	---	---
Mean Gradient, Catheter derived (mmHg)	1.3	3.2	5.0	5.9
Mean Gradient, Doppler derived (mmHg)	2.1	2.7	8.2	9.9
VED, first peak (Watts/meter)	0.22	0.32	0.84	1.39
First peak time (seconds)	0.03	0.04	0.47	0.48
VED, second peak (Watts/meter)	0.27	0.18	0.59	1.29
Second peak time (seconds)	0.49	0.48	0.54	0.54

MAC = mitral annular calcification, MVA = mitral valve area, VED = viscous energy dissipation

**Fig 6:**
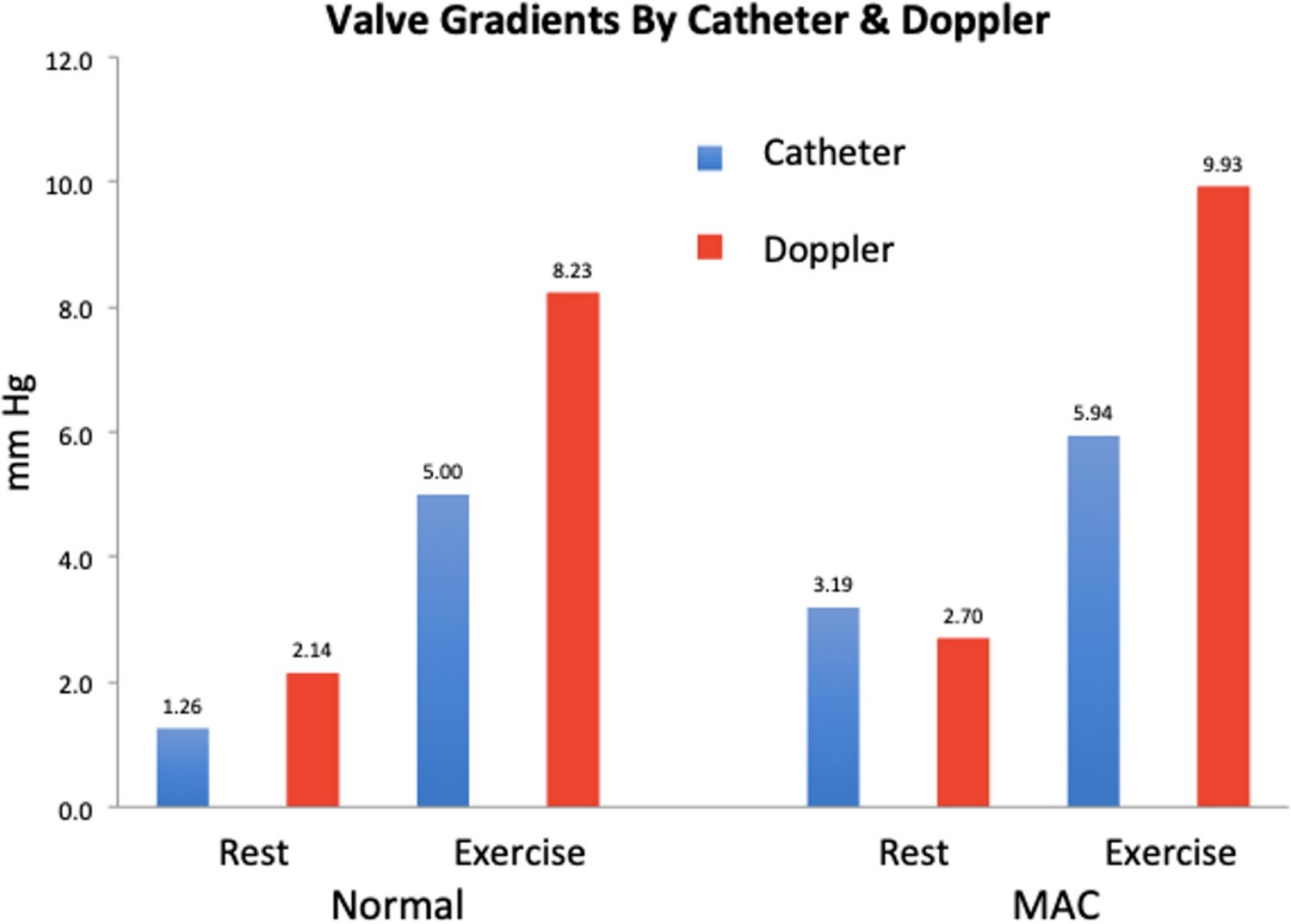
Pressure gradient assessment. Graphical representation of the pressure gradients assessed via echocardiography and catheter.

Under resting conditions, the catheter measured mean gradient across the normal valve was 1.3 mm Hg while the Doppler gradient was 2.1 mm Hg. For the severe MAC valve, the mean catheter gradient at rest was 3.2 mm Hg while the mean Doppler gradient was 2.7 mm Hg (Figs 5 and 6).

Under exercise conditions, the catheter measured mean gradient across the normal valve was 5.0 mm Hg while the Doppler gradient was 8.2 mm Hg. For the severe MAC valve, the mean catheter gradient for exercise was 5.9 mm Hg while the mean Doppler gradient was 9.9 mm Hg (Figs 5 and 6).

**Fig 7:**
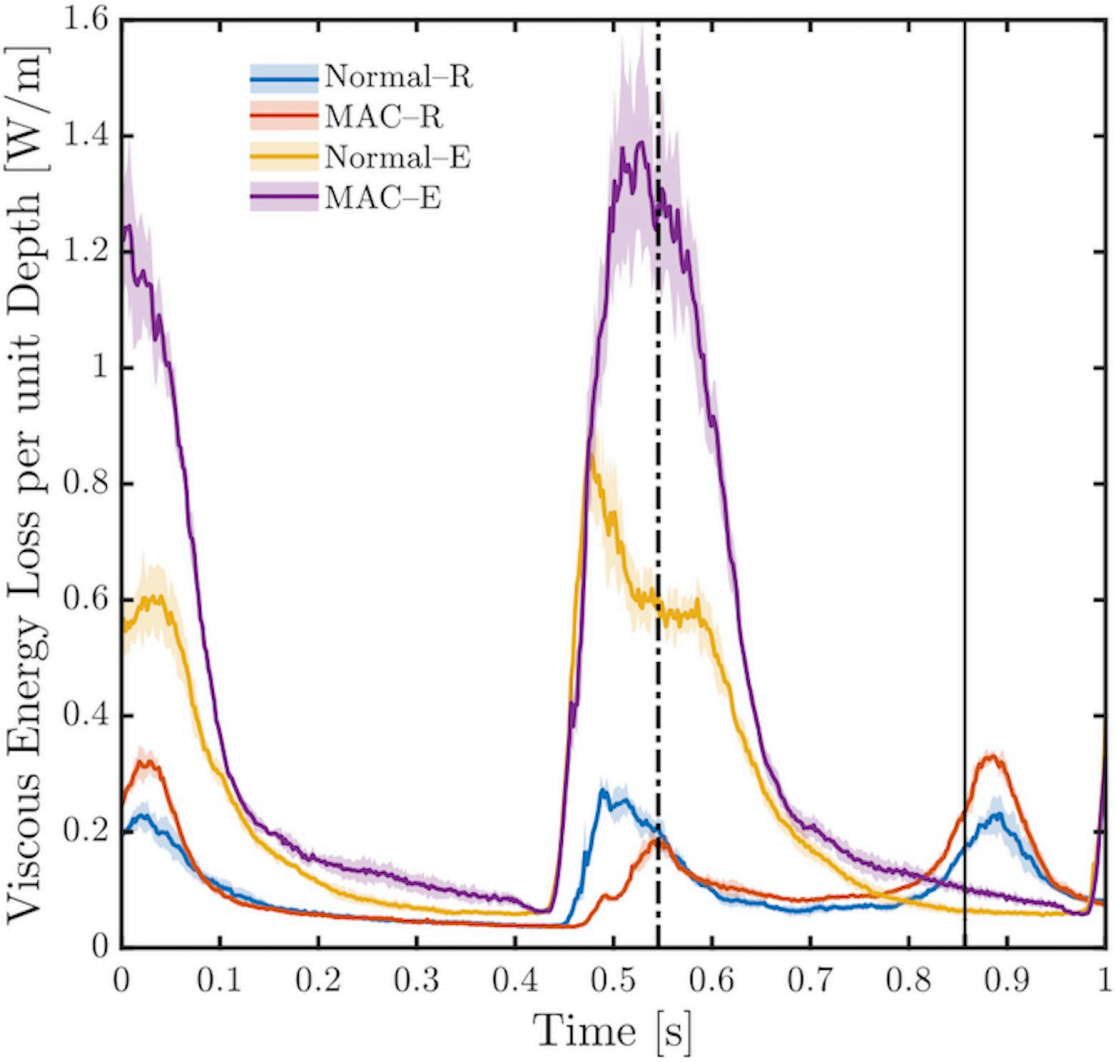
Viscous energy dissipation. Temporal evolution of viscous energy dissipation for the normal valve and for the severe MAC valve, both at rest and during exercise. The dashed-dotted vertical line indicates the end of the cardiac cycle during exercise, while the solid vertical line (between 0.8 and 0.9 on the x-axis) indicates the end of the cardiac cycle during rest conditions. The shaded area shows the upper and lower bounds of VED fluctuations for each case (between five recordings). The F-value (or measure of variance to signify dispersion) and p-value, both derived from ANOVA analysis between recordings for each case are as follow; Normal-R (0.79; 0.5327), MAC-R (0.83; 0.5046), Normal-E (0.86; 0.4859), MAC-E(0.59; 0.6677).

**Fig 8:**
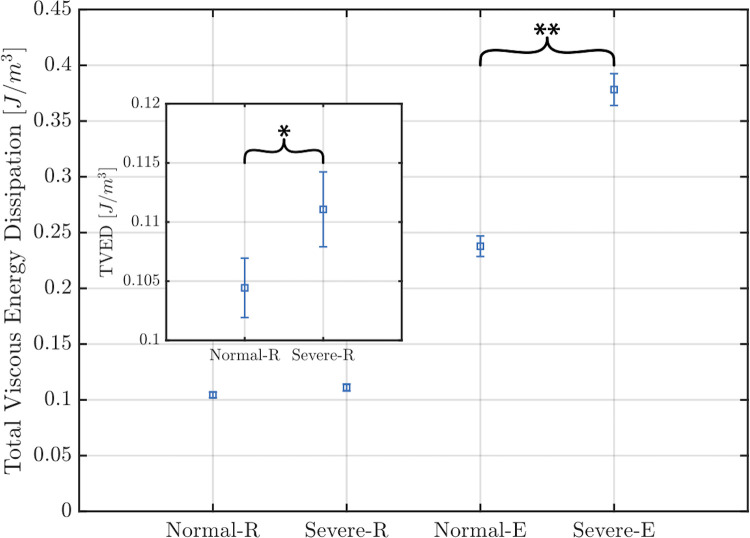
Total viscous energy loss. Mean value of total viscous energy loss computed for each case during 1 second including standard deviations. The insert magnifies the values for the normal and MAC valves under resting conditions. Normal-R = normal valve under resting conditions; MAC-R = MAC valve under resting conditions; Normal-E = normal valve under exercise conditions; MAC-E = MAC valve under exercise conditions. * represents a p-value = 0.0063, and ** represents a p-value = < 0.00001.

VED was calculated in the left ventricle for each valve under resting and exercise conditions (Figs 7 and 8; S3 Video). as in (1)

$$VED=\frac{\mu }{2}\int_{\forall i,j}{\left(\frac{\delta u_{i}}{\delta x_{j}}+\frac{\delta u_{j}}{\delta x_{i}}\right)}^{2}dA,$$ (1)

where *μ* indicates the dynamic viscosity of the fluid, *u*_*i*_ is the velocity component in x direction, *u*_*j*_ is the velocity in the y direction and d*A* is the interrogation window area.

In Fig 8, the temporal evolution of VED is displayed. For the normal valve under resting conditions, VED reached a first peak (0.22 W/m) at t = 0.03 s, where this peak represents the remaining effect of the A-wave. Shortly after the first peak, VED decays slowly during systole. A second peak (VED = 0.27 W/m at t = 0.49s) is noticed which corresponds to passive ventricular filling (the E-wave). Both peaks are consistent with the activation waveform in Fig 4. The reported VED values are in good agreement with those reported previously by Di Labbio and Kadem [15]. For the severe MAC valve under resting conditions, the first VED peak (remains of the A-wave) has a value of 0.32 W/m at t = 0.04 s, while the second peak (during the E-wave) has a value of 0.18 W/m at t = 0.54 s.

Under exercise conditions, the two VED peaks start to fuse. For the normal MV, during the E-wave, a sharp peak with a value of 0.84 W/m at t = 0.47 s is seen, while the semi-horizontal portion of the curve represents the beginning of the A-wave with VED = 0.59 W/m at t = 0.54 s. For the severe MAC valve, under exercise conditions, the maximum value of VED peak during the E-wave is 1.39 W/m at t = 0.48 s; moreover, the start of the A-wave can be identified by the VED peak with value = 1.29 W/m at 0.54 s. The remaining effect of the A-wave is noticed in the VED peak at t = 0.01 in the normal case and t = 0.05 s in the MAC case; in both cases this peak is followed by VED decay during ventricular systole.

Fig 8 shows the total viscous energy dissipation (TVED) for each valve under resting and exercise conditions. TVED represents the area under each curve in Fig 8 for a duration of 1 second. As can be appreciated from the graph, TVED was similar between the two valves under resting conditions. Under exercise conditions TVED rose by 127% for the normal valve and to a much greater extent (by 240%) for the MAC valve.

## Discussion

At present, standard measures for valvular disease assessment include transvalvular gradients, measured invasively and non-invasively, stress testing, and 3D-imaging. However, these measures provide an imperfect assessment of valvular function. We still frequently encounter patients who present late in the course of their disease and who go on to experience poor outcomes. We believe that VED is a more sensitive means of assessing the impact of valvular disease on cardiac function. In this research, we have successfully created and tested in vitro models of a normal MV and an abnormal MV with severe MAC. The major finding of this work is that VED increased more with exercise in the severe MAC valve as compared to normal. This occurred despite only minor differences in trans-valvular gradient between the two models. VED sums the energy losses at multiple points throughout the LV. Doppler measures gradient only along the line of interrogation and catheters reflect the average gradient for each chamber. Furthermore, while the gradient across the mitral valve provides information solely on the valve itself, VED in the LV provides useful information on the coupling between the mitral valve and the left ventricle. Thus, VED may detect abnormalities in the efficiency of blood transport through the heart that are not reflected in standard transvalvular gradient measurements.

It appears that severe MAC might lead to greater energy loss as blood is transported across the stenotic mitral valve, even when only a modest pressure gradient develops. VED is inevitable in the left ventricle, specifically during the filling phase where the mitral jet interacts with semi-stationary ventricular blood. In the normal situation, as the jet propagates inside the ventricle it follows a path close to the lateral wall that allows it to gradually energize the blood it encounters, minimizing the fluid strain rate and reducing VED. The situation is different with severe MAC, where the mitral jet is forced through a narrower orifice (caused by the MAC). This causes the transmitral flow to accelerate creating a higher rate of strain between the jet and the semi-stationary ventricular fluid (along the shear layer). Notably, the highly accelerated jet will propagate towards the apex while dissipating more viscous energy.

The observation of increased energy loss in the MAC simulation is of interest as many patients with MAC have limited exercise tolerance [17–19]. In addition, there are other clinical scenarios where vortex formation is disturbed leading to increased VED in the absence of a severe trans-valvular gradient. These scenarios include patients with prosthetic mitral valves where flow is often directed towards the ventricular septum as opposed to the normal situation where flow is directed more laterally [20, 21], and patients receiving a MitraClip device [22].

The other finding of interest relates to the discrepancy between catheter and Doppler gradients. Overall, the transvalvular gradients and valve areas compare reasonably well to those encountered clinically. Neither valve developed much of a gradient at rest, whether measured by catheter or by Doppler. Under exercise conditions, the catheter-based transvalvular gradient rose for both valves, with that of the MAC valve being slightly greater than for the normal valve. Interestingly, the Doppler measured gradients at exercise exceeded the catheter-based measurements to a clinically significant extent for both valves. This suggests that Doppler might overestimate the true transvalvular gradient for MAC valves under high flow conditions. The similar increase in Doppler gradient for the normal valve may relate to inherent stenosis in the model as evidenced by the valve area of 2.2 cm^2^. It should be noted that Doppler measured gradients have been validated for rheumatic stenosis [23], where the valve forms a funnel ending in a circular orifice. By contrast, MAC creates a tunnel-like stenosis at the level of the annulus with the commissures remaining open and the distal portion of the leaflets remaining freely mobile. Doppler measured gradients have not been validated in this situation and there are reasons to believe they may overestimate the true transvalvular gradient [24]. While this possibility needs further investigation, it is interesting to note that a previous *in vitro* experiment with a 3D printed MAC valve also observed higher gradients by Doppler when compared with catheter measured gradient [25]. Clinically, it has been observed that MAC patients have higher gradients, for the same valve area, than patients with rheumatic mitral stenosis [26].

This study has certain limitations. Because it was performed in vitro we cannot directly apply the results to patients due to the many simplifying approximations incorporated into our *in vitro* system. For example, the left ventricle is symmetric with a uniform contraction which deviates from the actual situation of the left ventricle. Also, the interaction between the left and right hearts was not represented in this model. Moreover, the MV chordae do not accurately duplicate the subvalvular apparatus. However, use of the *in vitro* setting allowed us to control all physiologic parameters and focus on effects of MAC. We chose to print the mitral valve in silicone and the MAC phantoms using PLA. These materials have similar physical properties to valve tissue and calcifications but do not precisely reproduce them. Computed tomography has better spatial resolution compared with TEE; it also allows for more precise delineation of calcifications. However, we chose to use TEE imaging for our 3D dataset as it has greater temporal resolution and provides more detail of the valve leaflets. As technology improves, we anticipate the ability to produce patient specific 3D-printed valves and cardiac structures. These could be tested in vitro to allow for personalized assessment of the physiologic impact of the mitral valve disease. Additionally, with the advent of commercially available ultrasound PIV systems (Vector Flow Mapping), we now have a clinical tool to measure energy loss in vivo [27–31].

In conclusion, VED in a 3D model of MAC mitral stenosis revealed marked differences under exercise conditions when compared with a control mitral valve. This contrasts with transmitral gradients which showed much smaller differences between the two valves. These findings may help explain the exercise related dyspnea and fatigue frequently experienced by patients with MAC.

## Supporting information

S1 Video Experimental testing of cardiac duplicator. Video clips of the normal valve (on the left) under rest conditions (demonstrated in real-time and slow-motion) and the severe mitral annular calcification (MAC) valve (on the right) during exercise conditions (in real-time).
(MP4)

S2 Video Ventricular view through mitral valve. A view from underneath the experimental apparatus; looking from the left ventricle toward the valve during simulated exercise for the MAC valve. To left is the mitral valve and to the right is the aortic valve. A light beam is cast above the left atrium and through the mitral valve to demonstrate valvular function. The calcium phantoms can be noted along the annulus.
(MP4)

S3 Video Particle image velocimetry. Particle image velocimetry (PIV) of a single cardiac cycle for the normal valve at rest compared with the severe mitral annular calcification (MAC) valve during exercise; note the distinct E- and A-waves during the cardiac cycle in the normal valve at rest and the blurring of E- and A-waves during exercise in the severe MAC valve.
(MP4)
